# Optical-Based Sensors for Monitoring Corrosion of Reinforcement Rebar via an Etched Cladding Bragg Grating

**DOI:** 10.3390/s121115820

**Published:** 2012-11-14

**Authors:** Muhammad Rosdi Abu Hassan, Muhammad Hafiz Abu Bakar, Katrina Dambul, Faisal Rafiq Mahamd Adikan

**Affiliations:** 1 Department of Electrical Engineering, University of Malaya, 50603 Kuala Lumpur, Malaysia; E-Mails: katrina@mmu.edu.my (K.D.); rafiq@um.edu.my (F.R.M.A.); 2 Wireless and Photonics Networks Research Centre, Universiti Putra Malaysia, 43400 Serdang, Selangor, Malaysia; E-Mail: mhab@ieee.org

**Keywords:** optical based corrosion sensor, etched fibre Bragg grating, Fibre Bragg grating, reinforcement rebar corrosion

## Abstract

In this paper, we present the development and testing of an optical-based sensor for monitoring the corrosion of reinforcement rebar. The testing was carried out using an 80% etched-cladding Fibre Bragg grating sensor to monitor the production of corrosion waste in a localized region of the rebar. Progression of corrosion can be sensed by observing the reflected wavelength shift of the FBG sensor. With the presence of corrosion, the etched-FBG reflected spectrum was shifted by 1.0 nm. In addition, with an increase in fringe pattern and continuously, step-like drop in power of the Bragg reflected spectrum was also displayed.

## 1. Introduction

Fibre optic sensors have a number of advantages over conventional sensors, yet they have not been widely adopted in civil engineering. Fibre Bragg grating (FBG) sensors offer a number of advantages over traditional sensors, including immunity to electromagnetic interference, light weight, small size, multiplexing capabilities, ease of installation and durability [1].

In the construction industry, reinforcement rebar can be corroded due to carbonation and chloride attacks [2]. Cracks are followed by spall that affects the durability of structures and if this problem is ignored, it can seriously corrupt the integrity of the structure, therefore periodical inspections are necessary, not only for the economic effects of corrosion damage, but also for safety concerns. As such, there is great demand for the development of non-destructive, *in-situ* and cost-effective techniques to provide early indication of corrosion without significant disassembly, thus reducing the cost of corrosion maintenance.

Greene utilized recoated long period gratings for real time detection of corrosion precursors and by-products for health monitoring and maintenance of aging aircraft [3]. Another method for sensing the degree of corrosion in concrete construction is to employ a vacuum plated sensitive film of reinforced steel bar placed on the core of the optical fibre [4]. Fuhr reported a fibre corrosion sensor for monitoring roadways and bridges [5]. He proposed to use a warning alarm in which a predetermined threshold of fibre events or faults can be set when detecting the structures internal damage. Grattan has applied Bragg sensors for sensing reinforcement bars in cement [6]. He used optical strain sensors with a cross comparison with the output of electrical resistance gauges to monitor the production of corrosion by products in concrete structures containing reinforcement bars.

This paper reports the development of an optical fibre corrosion sensor that detects corrosion based on the Bragg reflected wavelength spectra via an etched-cladding fibre Bragg grating. The development and characterisation of the FBG sensor for monitoring corrosion of reinforcement rebar were described from the early stages and throughout the entire corrosion event. The major aim of the work was to detect the wavelength shift and correlate this with the rate of corrosion in a localized region of the rebar.

Exposing the outer cladding of an FBG to certain stimulus such as corrosive environment will alter the effective refractive index of the composite cladding-substance structure. This will lead to a shift in the peak Bragg wavelength of the grating, which can be detected in several well-established ways. The technique which is known as evanescent field sensing has been proven in many applications including assessment of structural integrity [7] and oil pipe leakage [8]. By developing optical fibre sensors that can detect precursors to corrosion, the severity of the corrosive environment can be determined, leading to a more accurate maintenance system.

## 2. FBG Corrosion Sensor Sample Preparation and Experimental Setup

### 2.1. Fabrication of Etched-FBG

Meltz *et al.* proposed a sensing scheme for chemical sensing based upon Bragg grating in- and out-coupling for increased fluorescence excitation [9]. To increase the sensitivity of the surrounding index, a method of etching the optical fibre close to core diameter was presented in [10].

The principle of this sensor relies on the dependence of the Bragg resonance wavelength on effective refractive index and grating pitch. Our approach is based on the cladding-stripped Bragg uniform grating. If the fibre cladding diameter is reduced along the grating region, the effective refractive index is significantly affected by the external refractive index. The response of a Bragg grating is dictated by the known Bragg equation:

(1) $$\lambda_{\text{B}}=2n_{\text{eff}}\Lambda$$

where n_eff_ is the effective refractive index and Λ is the periodic spacing of the grating [11]. n_eff_ is a “weighted” average of two refraction indices. Exposing the outer layer of a waveguide with certain substances such as petrochemicals, will alter the effective refractive index of the composite waveguide-substance structure significantly. As a result, shifts in the Bragg wavelength combined with a modulation of the reflected amplitude are expected.

The technique to use is to immerse the Bragg grating into hydrofluoric (HF) acid while continuously measuring the Bragg reflection. A wavelength shift in the Bragg wavelength indicates that the evanescent field has begun interacting with the HF acid. A broadband light source, an optical circulator, and an optical spectrum analyser are used to monitor the wavelength shift.

The fibre grating was mounted in temperature compensated housing for fibre protection. The cladding of the fibre was initially etched to a diameter close to the diameter of the fibre to 25 μm for about 45 min in 48% HF acid solution. This first etch was controlled visually by measuring the diameter of the etched fibre under a microscope. The fibre was then etched controllably by measuring the Bragg wavelength as the fever was etched. The diameter of the final etched fibre is controlled by monitoring the reflection wavelength and peak power *in situ*. Since the etching speed of the Germanium doped core is faster than the silica cladding, the etching solution is then replaced with a 6:1 Buffered Oxide Etch (BOE), also known as buffered HF or BHF after 30 minutes. Using this *in-situ* monitoring, fibres with core diameters as small as 12−10 μm were produced with good precision.

### 2.2. Experimental Setup

**Figure 1.**
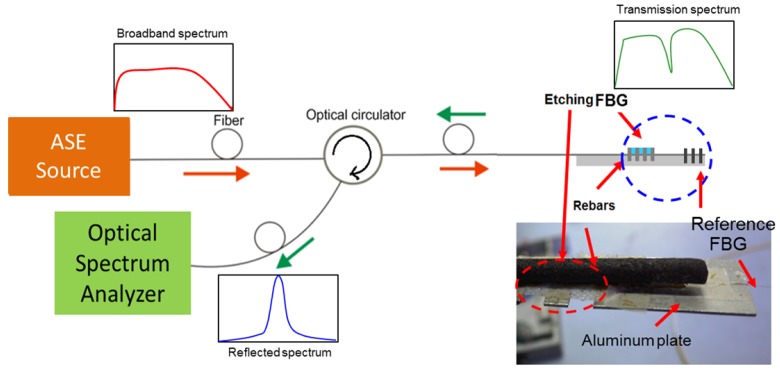
Schematic of an etched FBG sensor assembly to the rebar and image of the fibre sensor.

The experimental setup for measuring the Bragg wavelength shift is shown in Figure 1. A broadband erbium amplified spontaneous emission (ASE) light source was used. The spectral power of the ASE source was around 20 mW. The input signal passes through a circulator before being reflected by the FBG and directed to an optical spectrum analyser (OSA).

There is a need to have a protective mount for the fragile etched FBG. The design of the mounting of the FBG and the effect of glued on fibre has been discussed in [12]. The etched FBG used was mounted on the aluminium plate as shown in Figure 1. For this work; a steel rebar measuring 10 mm diameter by 750 mm long was used. The rebar was exposed to salt water to induce corrosion. In this test, the direct gluing of the sensor to the metal plate could inhibit corrosion of the bar directly beneath the sensor. Nevertheless, it has been shown that shifted Bragg wavelength spectra will still be recorded. As reference, a non-cladding etched FBG sensor was fixed at one end of the same rebar for temperature and strain compensate as shown in Figure 1. The temperature can be measured and compensated for by using a second grating element and placed in series with the first grating [13]. The wavelength shift readings for both sensors were recorded and examined for any variation arising from the corrosion process.

## 3. Results and Discussion

**Figure 2.**
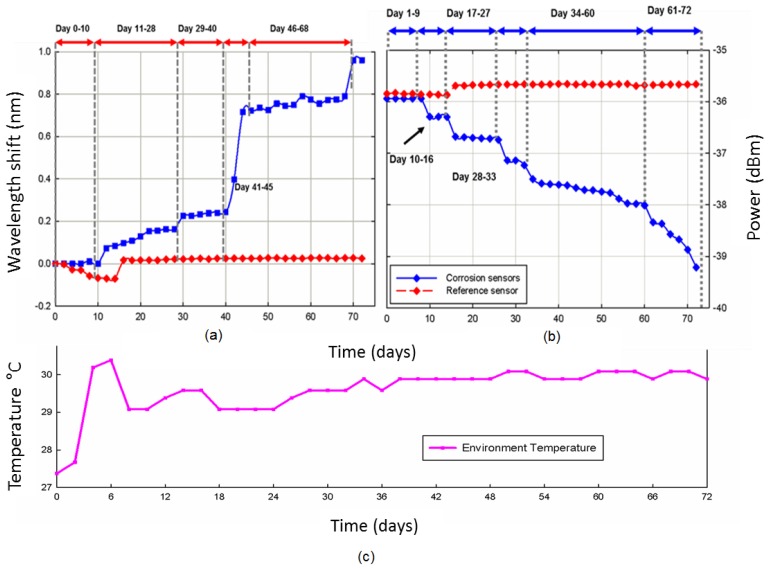
(**a**) The Bragg wavelength shift with time (days). (**b**) The peak power (dBm) with time (days). (**c**) The tempreture fluctuation of environment condition during experiment conducted.

Figure 2(a) shows the wavelength change with time (days) from both sensors. The experiment started on 4 November 2011 and for the first ten days, there was no wavelength shift observed from the FBG sensor. After day 10, the rusting due to corrosion of the steel was visually evident and the Bragg wavelength starts to shift by about 0.07 nm. From day 11 to day 28, the wavelength shift reading kept increasing gradually until day 40. The correlation between corrosion and the central reflected wavelength of FBG, λ_B_, is defined by what is known as the Bragg condition Bragg equation as in Equation (1).

In our case, the steel interacted with the salt water and their caused a breakdown of the passive film on the surface of the rebar. This allowed rust to form that subsequently lowered the pH level and created an acidic environment around the rebar. The changes in the environment around the fibre introduced variations to n_eff_ and resulted in a shift in the central reflected wavelength. Through calibration, this shift can be related back to the parameter under investigation, in this case the corrosion rate of the rebar.

As an overall trend, it is clear that the wavelength shift increases steadily at approximately 0.2 nm between days 0 to day 40. Beginning from day 41, we can observe a rapid rise in wavelength shift that went up to 0.72 nm by day 45. This is due to increased corrosion activity in the metal rebar that can be attributed to higher humidity caused by heavy rain in the month of December 2011 as reported by the Malaysian Meteorological Department [14]. From day 46 onwards, the wavelength shift reached a plateau, which was sustained until day 69 at 0.8 nm. In day 70, the FBG sensor was reached the maximum peak at approximately 1.0 nm. In the experiment, The FBG sensor provided reading and was still functional until the end of the test on day 74. In contrast, the wavelength shift of reference FBG remained constant without any shift throughout the experiment. It shows that the environment temperature does not influence the experimental result. The strain change will also be induced by corrosion; however, we consider the response of strain was assumed to be minimum significant compare the changes of refractive index of FBG during the corrosion occur.

As we can see in Figure 2(b), the reading of spectral power also sees a continuous drop on the range of that day. On day 0, the spectral power starts with −35.922 dBm. By day 10, the readings of spectral power drop to −36.286 dBm and decrease more steeply to −37.639 dBm on day 29. The spectral power continues to decline gradually and had dropped to −39.309 by day 70. There was no change in spectral power for reference FBG except for a small rise in spectral power on day 16. Figure 2(c) shows the temperature fluctuation on the reference FBG. It demonstrates that the influence of environment temperature is controlled and has minimal influence on the reading of Bragg wavelength shift.

**Figure 3.**
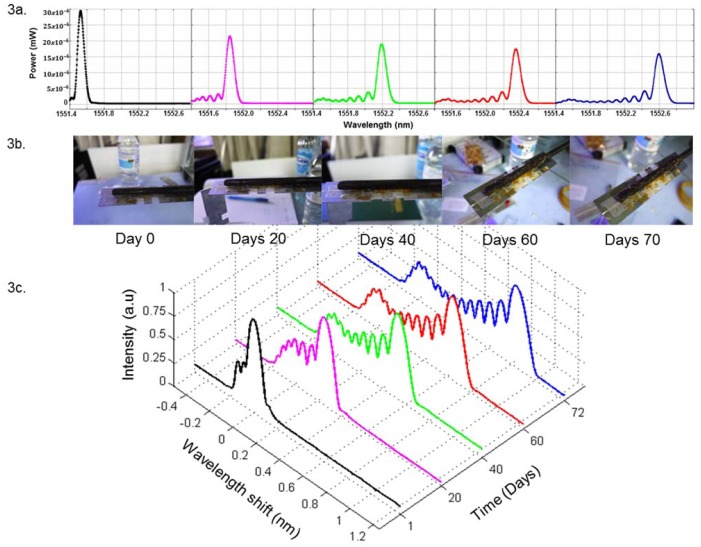
Reflected spectra of FBGs broadening as the corrosion of metal rebar progress.

It can be clearly seen that the spectral power fell consistently from a starting point of nearly 30 × 10^−6^ mW on day 0 to less than 15 × 10^−6^ mW on day 70. The significance of corrosion progress of the metal rebar through the experiment was evident, as shown in Figure 3(b). Figure 3(c) shows the reflected spectrum of FBGs, exhibiting significant broadening as corrosion increases during the experiment. The spectral broadening is associated with Fabry-Perot type modulation due to corrosive gradient induced chirping of the grating [15]. The fringes on the Bragg reflected spectrum, is evident. We believe this is due to localized corrosion along the length of the Bragg, leading to different n_eff_ values along the Bragg. This produces a chirping effect, resulting in fringes. This effect is due to the decrease in the effective refractive index induced by the interaction of the solution with refractive index lower than the cladding, so as the Bragg reflected spectrum shift to the left of the fringes is larger and more apparent.

## 4. Conclusions

We have proposed and demonstrated experimentally an FBG sensor for corrosion monitoring. The sensor consists of an etched FBG mounted on an aluminium plate. It is able to detect the production of corrosion waste. The Bragg wavelength will shift to the right to the presence of corrosion. This approach offers a real-time, fast, *in-situ* and inexpensive technique for applications involving remote monitoring of corrosion on civil structures exposed to corrosive environments.
